# Machine Learning for Endometrial Cancer Prediction and Prognostication

**DOI:** 10.3389/fonc.2022.852746

**Published:** 2022-07-27

**Authors:** Vipul Bhardwaj, Arundhiti Sharma, Snijesh Valiya Parambath, Ijaz Gul, Xi Zhang, Peter E. Lobie, Peiwu Qin, Vijay Pandey

**Affiliations:** ^1^ Tsinghua Berkeley Shenzhen Institute, Tsinghua Shenzhen International Graduate School, Tsinghua University, Shenzhen, China; ^2^ Division of Molecular Medicine, St. John’s Research Institute, Bangalore, India; ^3^ Institute of Biopharmaceutical and Health Engineering, Tsinghua Shenzhen International Graduate School, Tsinghua University, Shenzhen, China; ^4^ Shenzhen Bay Laboratory, Shenzhen, China

**Keywords:** machine learning, endometrial cancer, artificial intelligence, deep learning, histopathology, prediction model, algorithm

## Abstract

Endometrial cancer (EC) is a prevalent uterine cancer that remains a major contributor to cancer-associated morbidity and mortality. EC diagnosed at advanced stages shows a poor therapeutic response. The clinically utilized EC diagnostic approaches are costly, time-consuming, and are not readily available to all patients. The rapid growth in computational biology has enticed substantial research attention from both data scientists and oncologists, leading to the development of rapid and cost-effective computer-aided cancer surveillance systems. Machine learning (ML), a subcategory of artificial intelligence, provides opportunities for drug discovery, early cancer diagnosis, effective treatment, and choice of treatment modalities. The application of ML approaches in EC diagnosis, therapies, and prognosis may be particularly relevant. Considering the significance of customized treatment and the growing trend of using ML approaches in cancer prediction and monitoring, a critical survey of ML utility in EC may provide impetus research in EC and assist oncologists, molecular biologists, biomedical engineers, and bioinformaticians to further collaborative research in EC. In this review, an overview of EC along with risk factors and diagnostic methods is discussed, followed by a comprehensive analysis of the potential ML modalities for prevention, screening, detection, and prognosis of EC patients.

## Introduction

Rising endometrial cancer (EC) incidence and disease mortality represent a serious concern for women, particularly in countries with rapid socioeconomic transitions where the incidence rate of this cancer is the highest (1–3). The International Federation of Gynecology and Obstetrics (FIGO) staging method is used to determine the surgico-pathological staging of EC (4). The majority of EC patients are diagnosed at an early stage (80% in stage I), with a 5-year survival rate exceeding 95%, the highest of all gynecological cancers (5). Individuals with early detection or who have EC with a reduced risk show a favorable prognosis. Individuals detected with higher stage EC who have developed recurrence exhibit a worse 5-year survival ranging between 47% and 58% for stage III EC patients and 15% and 17% for stage IV EC patients; and possess a limited number of accessible prognostic or therapeutic alternatives (6, 7). Expensive screening, and a high rate of misdiagnosis majorly contribute to high disease mortality (8–10). Although endometrial biopsy with dilation and curettage is the standard diagnostic approach for EC, there exist no clinically validated EC screening approaches (11). Progestin treatment is appropriate for women experiencing atypical endometrial hyperplasia (AEH), a precancerous type of endometrial lesion, or stage 1A EC lacking muscle penetration (12). Most women diagnosed with EC exhibit a good prognosis after surgery alone; however, poorer outcomes are associated with high-grade, recurrent, and metastatic EC (13). Therefore, routine screening, early detection, and precise prediction of recurrence or survival after oncotherapeutic regimens may improve the survival of EC patients, rather than a simple presentation of symptomology. In this review, machine learning (ML)–based strategies and techniques that could improve the prediction and prognostication of EC are discussed.

ML approaches (algorithms) have evolved in oncology to raise the reliability of predication of cancer susceptibility, recurrence, and survival (14, 15). ML is a subfield of artificial intelligence (AI) that combines a range of statistical, probabilistic, and optimization techniques to enable computers to “learn” from previous examples and detect complicated patterns in vast, noisy, or complex datasets (16, 17). AI allows computers to execute “cognitive” tasks for humans, such as language comprehension, reasoning, and problem solving. The use of an appropriate AI system enables computers to discover patterns in available datasets and derive inferences using the data without requiring explicit commands (18). Currently, AI has mostly been utilized for image identification tasks in healthcare (19). Several articles have reported the high accuracy of AI in diagnosing conditions such as skin cancer, and diabetic retinopathy (20–23). ML algorithms have been effectively employed in the treatment of cancer, such as breast cancer (24), oesophageal cancer (25), head and neck cancer (26), osteosarcoma (27), prostate cancer (28), and thoracic cancer (29). ML offers the opportunity to “systematically evaluate every variable, present, and future, to locate groupings of cancer cases with similar outcomes” as cancer prediction and prognostication systems become more complicated with rising variables. Implementation of ML approaches for EC prediction and prognostication should be of utility as patients with diverse outcomes may be subcategorized into specific clinical stages.

## Clinico-Pathological Features of EC

EC has typically been classified into two types: type I and type II (30). These two classifications differ in terms of epidemiology, histology, prognosis, and treatment (30, 31). Type I EC is the most common form and accounts for most diagnosed cases (80%), has an overall 5-year survival rate of 81.3%, and usually has less than 20% chance of recurrence (31, 32). Type I EC offers a good prognosis in the majority of patients because they are low grade and limited to the uterus at the point of diagnosis (33). Type I EC is predominantly associated with obesity-related complications and with excessive endometrial cell proliferation (34). Type I EC is also observed to be susceptible to excessive exposure to estrogen, *via* both endogenous and exogenous routes and mainly affects younger women (premenopausal or perimenopausal) (33). As a result, hyperestrogenism, hyperlipidemia, diabetes, and anovulatory uterine bleeding are common in individuals with type I EC. Hence, pathological conditions that are associated with metabolic deregulation have been recognized as an autonomous risk component for the early onset of the EC (35). Endometrial intraepithelial neoplasia (often termed complex AEH) is a type I EC precursor lesion that is frequently associated with a thicker endometrium and shares the same estrogen exposure risk factors as EC (36, 37). Hyperplasia contributes to a 1%–3% risk of the development of cancer. Low-grade endometrioid adenocarcinomas [International Federation of Gynecology and Obstetrics (FIGO) grades 1 and 2] are the most common type I cancer in women (38). Grade 1 cancers are well differentiated, resemble normal tissue, and often show favorable prognosis (39). Grade 2 malignancies contain a solid component that ranges from 6% to 50% and are classified as differentiated. Grade 1–2 ECs are also classified as type I; grade 3 tumors that contain a solid component ranging >50% are high grade and poorly differentiated, and they do not appear as normal endometrial tissue, are aggressive, and associated with poor prognosis. Grade 3 EC is classified as type II EC, often affects old age women (postmenopausal), and is not associated with endocrine disorders (40). Type II cancers are high-grade, non-endometrioid histology and are mostly composed of serous carcinomas and clear cell carcinomas (41). Type II ECs are frequently diagnosed at a late stage, associated with intermediate-to-poor prognosis, and a high rate of recurrence with a decreased 5-year overall survival rate (55%) which contributes disproportionally to disease mortality (31). Although type II ECs contribute to only 20% of all ECs, they are associated with ~40% of all EC patients with poor overall survival (42). Around 20% of endometrioid cancers are subcategorized as high grade (FIGO grade 3) and type II EC (31). Dedifferentiated, undifferentiated, mixed cell, neuroendocrine, and carcinosarcomas (also known as malignant mixed mullerian tumors) are similar to serous and clear cell carcinoma histology. Type II EC is more prevalent in elderly women and is especially frequently observed in African-American women with thin and atrophic endometrium (13, 21).

Increased risk of developing type I EC, is related to unopposed exposure of the endometrium to estrogen (E2) (43). Hormone replacement therapy represents an example of exogenous estrogen exposure (44). Premature menarche, late menopause, tamoxifen treatment, nulliparity, infertility or inability to ovulate, and a polycystic ovarian disease can all increase uterine estrogenic exposure (45). Family history, age (over 50), hypertension, diabetes mellitus, obesity, and thyroid disease are all independent risk factors through which the risk of EC increases (33, 42, 46, 47). Being obese is the most significant risk factor for hyperplasia progression to malignant EC (48). Obesity affects more than 70% of people with initial stage EC (49). Obesity is also hypothesized to increase the risk of EC by increasing the peripheral conversion of androgens to estrone in adipose cells (39). Obese premenopausal women are also highly susceptible to prolonged anovulation, which is an additional risk factor for EC (48). Genetic conditions like Cowden syndrome, Lynch syndrome, and polymerase proofreading-associated polyposis are associated with an increased risk of developing EC among women (50, 51). Lynch syndrome is a cancer risk disease characterized by a monoallelic germline mutation in a mismatch repair (MMR) gene, particularly MLH1, MSH2/6, or PMS2, or by a germline deletion inside epithelial cell adhesion molecule (EpCAM) that contributes to epigenetic silencing of the neighboring MSH2 gene (52). Carriers of these mutations are more likely to develop ECs (53).

Early detection of EC can improve the chances of a good prognosis. Abnormal uterine bleeding and postmenopausal vaginal bleeding (PMB) are categorized as the most prevalent symptom of EC. Despite the fact that PMB is present in 90% of women with EC (regardless of tumor stage), it is not a reliable indicator of the disease. Only 9% of PMB patients are diagnosed with EC (54). Internal pelvic examination with a Pap (Papanicolaou) smear test is generally regarded as the initial investigation when symptoms, signs, and/or family history imply the existence of gynecologic pathology (55). However, the Pap smear is not a useful predictor of EC and is predominantly utilized for cervical cancer screening and detection (56). Transvaginal ultrasonography (TVUS) is the most helpful tool to use in gynecologic practice to monitor the female reproductive organs as it can help to determine the thickness and features of the endometrial lining, as well as the size of the uterus, the adnexa, and the presence of excess pelvic fluid (57). The TVUS probe (which acts similarly to an ultrasound transducer) is inserted into the vagina for the transvaginal scan (TVS). Images from the TVS are then utilized to determine if there is a mass (tumor) in the uterus or if the endometrium is thicker than normal, which could indicate EC. It is also used to determine if cancer has spread to the uterine muscular layer (myometrium) (58). As a triage tool, TVS-based endometrial thickness screening lacks sufficient specificity because it cannot distinguish benign lesions, such as polyps, from their malignant counterparts exposing a large proportion of women to further testing (59–61). The endometrial histological information provided by endometrial biopsy is a gold standard for diagnostic evaluation and sufficient for preoperative assessment (62, 63). In combination with EC biopsies, dilation and curettage (D&C) are often recommended to confirm the EC diagnosis; however, the D&C method is painful, expensive, requires general anesthesia and has a high rate of misdiagnosis in up to 31% of women and demands multiple repeats for confirmation (8–10, 64). Another technique used to investigate EC is hysteroscopy, which allows for direct viewing of the endometrial cavity, which is often used to examine abnormal uterine bleeding (42). Hysteroscopy can be combined with a focused biopsy or curettage. In the detection of EC, hysteroscopy yields higher accuracy than does blind D&C and had a sensitivity of 99.2% and a specificity of 86.4% (65). Thus, except for histology of endometrial biopsies, there is no clinically accepted method for screening, detection, prediction, and diagnosis of EC.

To determine local extension and any metastatic disease, imaging studies such as magnetic resonance imaging (MRI), computerized tomography (CT), or positron emission testing/CT may be used. However, imaging studies are limited in the detection of lymph node dissemination, which is observed in at least 90% of the cases using microscopic-based approaches (66). However, one of the more interesting and difficult challenges for clinicians is accurately predicting the outcome of an illness. As a result, research is increasingly employing ML-based approaches that are capable of discovering disease-associated patterns and links in the large datasets and may accurately predict potential disease risks and outcomes for individual patients.

## Machine Learning: Methods and Algorithms

**Figure 1:**
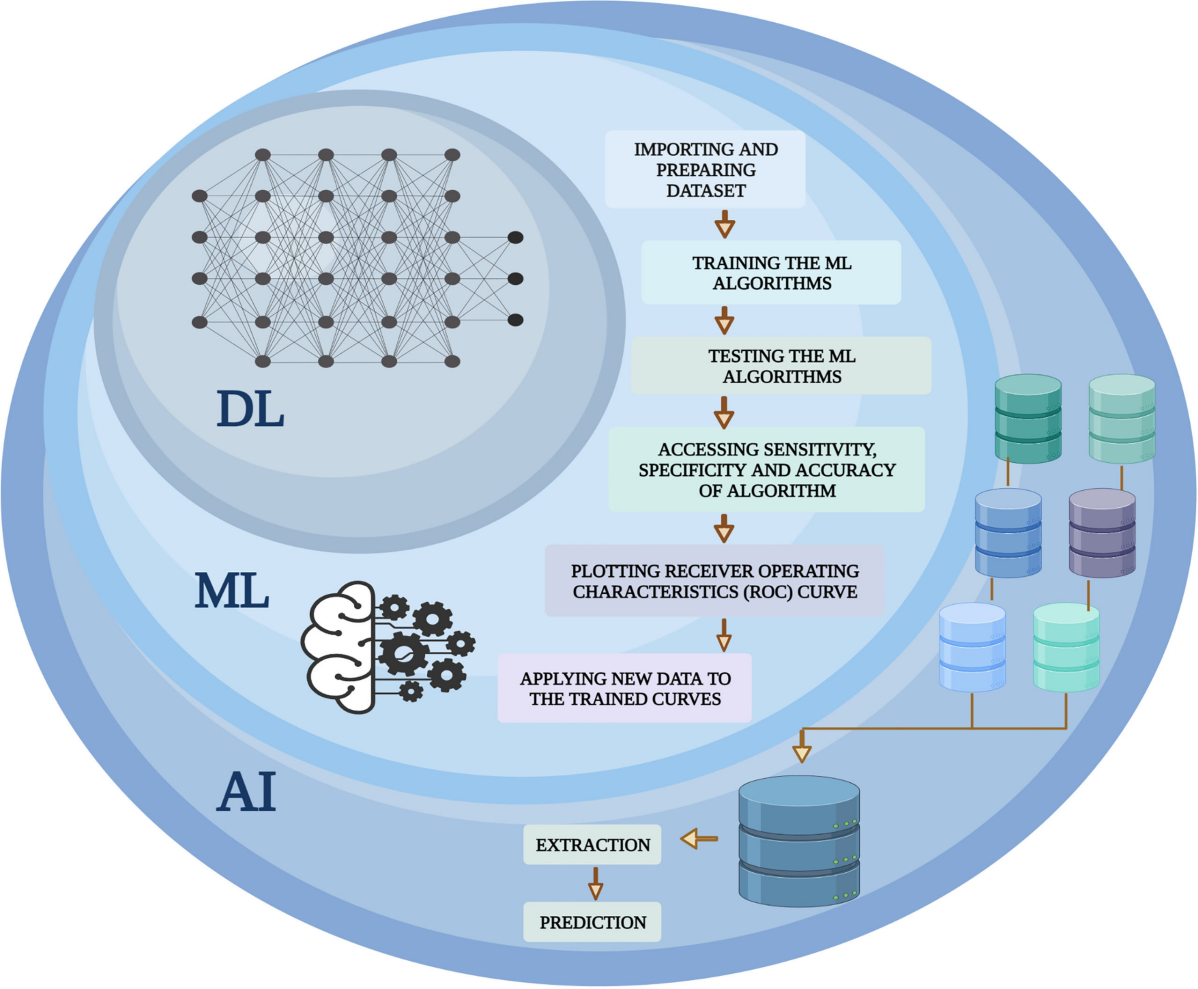
An overview of the machine learning (ML) integration with artificial intelligence (AI) and deep learning (DL). A computer science branch that uses machines and programs to mimic human intelligence is known as AI, whereas DL is a subgroup of AI that employs models from statistics and mathematics. ML covers a variety of algorithms and statistical methods, including logistic regression, random forest, and DL-based approaches. The ML algorithm is continually integrated with a new dataset to test the validity and utility of algorithms.

In 1959, Arthur Samuel first coined the expression “machine learning”. ML determines a machine’s capability to understand and simulate upcoming scenarios and potential effects predicated upon massive datasets. Hence, the science of having a machine function learning from the data, recognizing patterns, and giving the outputs with minimal human input is ML. It has brought to society autodriven vehicles, functional comprehension of voice, robust online search, and a dramatically enhanced understanding of the human genome. ML is a bustling field of medicine with tools being used to integrate medical challenges with computer science and statistics. ML may lead to more detailed diagnostic algorithms in medicine and personalized patient care. ML is a data mining software for creating analytical models that is fully automated and is a subset of AI-based on the notion that machines could extrapolate data, interpret trends, and generate results with little human input. Data samples contain the basic constituents that are required to develop a strategy for the application of the ML algorithm. Sample representation contains multiple features and each function consists of numerous classification values. Understanding the particular form of data that is used in advance facilitates the proper collection of methods and algorithms that could perhaps be employed for the evaluation. Compared with conventional biostatistical approaches, the strengths of ML comprise versatility and scalability, which make it possible for multiple functions such as stratifying threats, diagnosing and classifying, and predicting survival. A further value of ML algorithms is to integrate different forms of data (e.g., population data, experimental outcomes, and data from imaging) to identify patterns that could effectively classify the data into respective categories. Amid such benefits, the use of ML in health care poses specific difficulties that include preprocessing of data, experimental design, and algorithm refinement related to the specific clinical issue (67). A comprehensive overview of AI and its subfields [ML and deep learning (DL)] is summarized in **Figure 1**.

**Figure 2:**
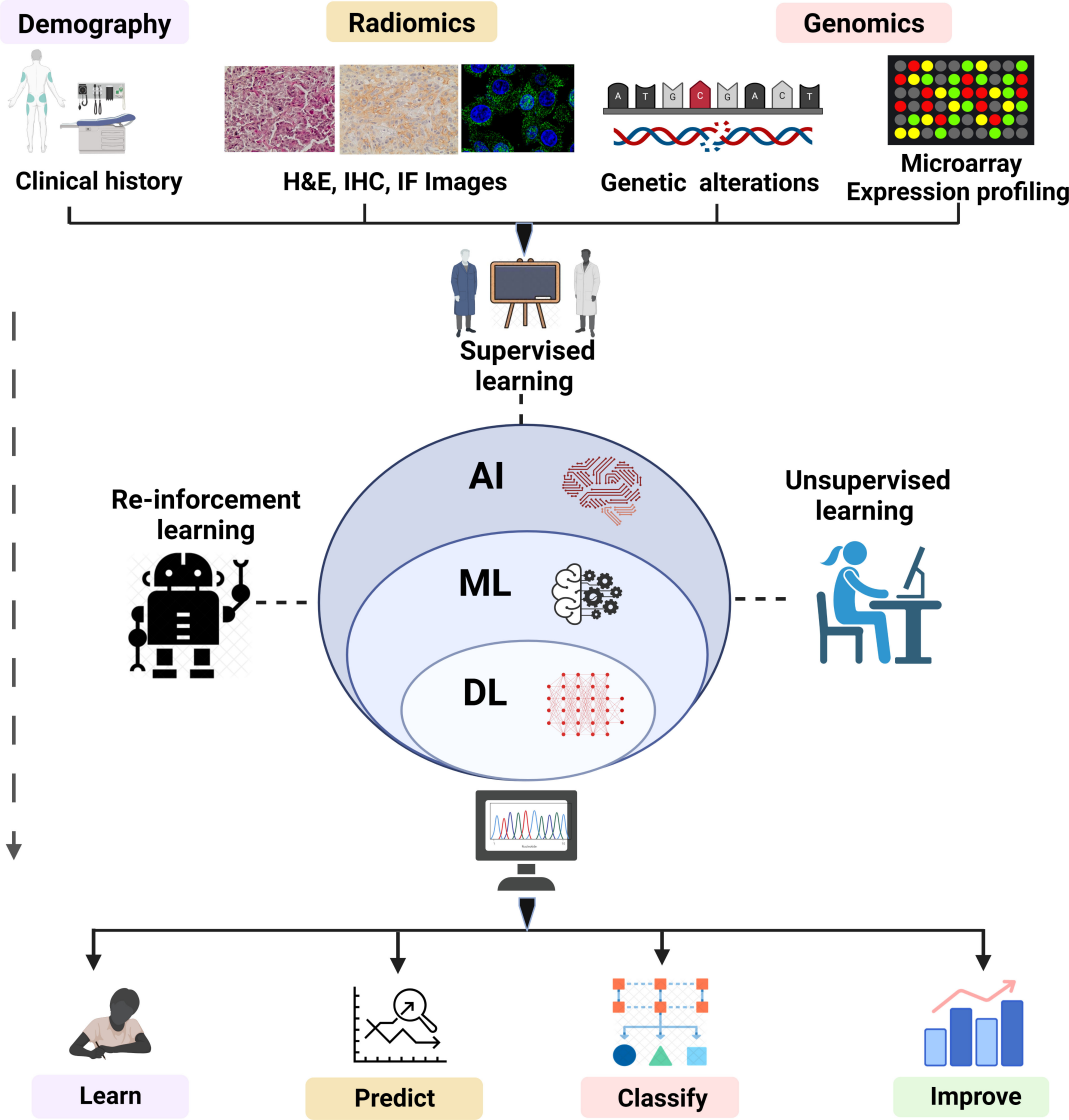
Overview of ML (supervised, unsupervised and reinforcement learning). The overview of ML depicts the analysis and testing of statistical models and algorithms that computational approaches used to perform a specific task without being explicitly programmed. The figure represents subdisciplines of AI (ML and DL) and their subtypes including supervised, unsupervised, and reinforced algorithms employed in fields such as pattern recognition, object detection, text interpretation, and genomics. The algorithms of ML learn, improve, predict, and classify the data.

ML plays an instrumental role to speed digital transformation and is ushering in an age of automation. ML has become so prevalent that it has now become one of the preferred methods for researchers to handle a wide range of biological problems. The emergence of computer-aided systems that have been instructed to perform complex tasks in medical imaging, bioinformatics, and medical robotics has stemmed from the accessibility of advancing computational power, strongly enhanced pattern recognition algorithms, and enhanced image processing (IP) software operating at incredibly fast acceleration. A “cognitive” computer having exposure to “big data” may scan billions of bits of unstructured data, retrieve user data, and detect complicated patterns with growing confidence. Several ML algorithms are mathematical models that transfer a collection of observable variables through a data point or sample, referred to as “features” or “predictors”, into a set of outcome variables, referred to as “labels” or “targets” (68, 69). Widely used ML algorithms with their advantage and limitations are shown in **Table 1**. The algorithms are trained to be competent to anticipate labels by analyzing specific information in a phase termed “training”. Presently, three prominent methods are used to train ML algorithms: supervised, unsupervised, and reinforcement learning (**Figure 2**).

**Table 1:** List of algorithms used in ML with the advantages and limitations.

ML Algorithms	Advantages	Limitations
Decision Tree (70)	- Training method that is simple to comprehend and efficient - Training is unaffected by the sequence of training occurrences. - Pruning reduces the complexity of the classifier and improves predictive accuracy by the reduction of overfitting	- Classifications must be mutually exclusive - The final decision tree is determined by the order in which the algorithm parameters are selected - Inaccuracies in the training set might lead to excessively complicated decision trees - When attribute’s values are missing, it is uncertain which branch to choose when that attribute is checked.
Naïve Bayes (71)	- Statistical modeling–based basis -Training method that is simple to comprehend and efficient - Training is unaffected by the sequence of training occurrences - Useful in a variety of accuracy areas.	- Presupposes that those characteristics are statistically independent - Expects that numeric characteristics have a normal distribution - Classifications must be mutually exclusive - Redundant characteristics classification error - Attribute and class frequencies have an impact
k-Nearest neighbor (72)	- Cases are quickly classified. - Beneficial in non-linear classification situations - Resilient in the face of irrelevant or new features - Capable of withstanding noisy instances or instances with missing attribute values - May be used for regression as well as categorization	- Implies that instances with identical characteristics will be classified similarly - Believes that all characteristics are equally important - When the number of characteristics grows, it becomes too computationally complex
Neural Network (73)	- It has the potential to be utilized for classification or regression. - Capable of representing Boolean functions (AND, OR, NOT) - Tolerable to loud input - More than one output can be used to classify instances	- The algorithm’s structure is tough to grasp - Overfitting can occur when there are too many characteristics - Experimentation is the only way to discover the best network structure
Support Vector Machine (74)	- Nonlinear class boundaries are modeled - It is unlikely that overfitting will occur - Decreased computational complexity to a quadratic optimization issue - It is simple to adjust the complexity of the decision rule and the frequency of mistake	- Compared to Bayes and decision machine trees, training takes longer - Finding optimum settings is difficult when training data are not linearly separable - The algorithm’s structure is difficult to understand
Genetic Algorithm (75)	- Simple algorithm that is simple to implement - Can be utilized for feature categorization and selection - Utilized mostly in optimization - Always comes up with a “decent” answer (not always the best solution)	- It is difficult to compute or create a scoring function - It is not the more productive approach for locating some optima since it prefers to discover local optima instead of global selection - Complications involved in the representation of training/output data

## Imbalanced Data in ML

ML algorithms are powered by the volume of data in datasets. A balanced dataset is one in which the distribution of labels is approximately equal. Labels, in this case, indicate the class related to each data point. The class label is projected by evaluating the input data or predictor in a classification issue when the target or output variable is a categorical variable. A class imbalance is common in most classification issues. In certain cases although, when the dominant class is much bigger as compared to the minority class, the disparity is quite pronounced. They occur when one of the target class labels has a significantly lower number of observations than the other class labels. An imbalanced class dataset is a type of dataset that is particularly common in real classification scenarios (76). Any conventional strategy to solve this type of machine learning challenge frequently produces ineffective results. In unusual situations like fraud detection or disease prediction, it is vital to correctly determine the minority classes. As a result, the model should not be biased toward recognizing just the majority and therefore must assign the minority class the same relevance or value as the majority. Most machine learning algorithms, on the other hand, struggle with imbalanced datasets (77). When dealing with imbalanced datasets, there is no one-stop solution to increase the predictive accuracy of a model. It may be required to use several processes to determine the best sampling methodology for the dataset. The most successful strategies will differ depending on the peculiarities of the imbalanced data set (). Resampling strategies, ensemble learning techniques (78–80), use of right evaluation metrics, boosting, cost-sensitive learning (81), one-class learning (82), and active learning have all been considered as solutions to the class imbalance problem. In resampling strategies, the most commonly employed methodologies to assess the class imbalanced issues are by using either random oversampling or random undersampling.

## ML Application in EC Prediction, Diagnosis, and Prognosis

The advanced stage of diagnosis and limited therapy options seriously hinders the prognosis of EC patients. Several investigations have suggested that screening, early detection, monitoring, and prediction of EC could significantly improve the prognosis of patients. Advances in ML techniques offer unique and promising perspectives for the detection and prediction of several cancers such as breast, colorectal, and prostate cancer. Lately, ML has had a significant impact on the development of potential computational tools for stratifying, scoring, and prognosticating cancer patients to improve patient survival (12). Recent studies have reported that ML algorithms have been utilized to identify lymph node metastases, scoring KI67 positivity in breast cancer, scoring tumor-infiltrating lymphocytes in melanoma, and Gleason grading in prostate cancer (83). ML has also successfully attempted to predict tumor recurrence with high accuracy using pathological images (84). Furthermore, Pariss et al. used a novel ML-based algorithm to demonstrate an improved prognostic prediction for patients with EC (85). Thus, ML-based approaches can be employed to maximize the sensitivity and specificity of EC diagnosis and prognosis.

### ML Approaches in Image and Pattern Recognition

Pattern recognition is the process of automated distinguishing, recognizing, and segmenting patterns and regularities in data using ML algorithms. It categorizes data using statistics, mathematical models, or knowledge derived from patterns and their representation. In supervised pattern recognition, the data are trained using specific labels. A label is assigned to each input value that is utilized to generate a pattern-based output. On the other hand, in unsupervised pattern recognition, various computer algorithms like clustering or principal components analysis are used to detect unknown patterns in the absence of labeled data. IP is a type of computer technology that allows us to process, analyze, and extract data from pictures. Some studies applying machine learning patterns and image recognition algorithms in EC have been summarized below.

There has been substantial progress in the application of pattern recognition and IP in the detection, classification, and identification of EC. Attempts have primarily been made in medical imaging to incorporate AI in preoperative diagnostic tools such as endoscopy, CT, MRI, ultrasound imaging, and pathological imaging. MRI is an essential medical imaging tool assisting in the identification and preoperative assessment of EC patients, and there have been some reports on its use in conjunction with DL approaches (86–88)]. Hodneland et al. demonstrated a fully automated approach for tumor segmentation in EC using a 3D convolutional neural network (CNN) named UNet3D (89) applied to a cohort of 139 EC patients with preoperative pelvic MR images (86). The whole value of tumor texture features and tumor volume estimations along with the tumor segmentation accuracy was obtained. The study showed that the available ML algorithms may offer accurate tumor segmentation at the level of a human expert in EC. The model generates tumor volume, tumor borders, and volumetric tumor maps. Hence, the self-generated approach for primary EC tumor segmentation seems to exhibit the prospect to seek near-real-time whole-volume radiomic tumor profiling, including tumor volume and texture properties, which could be useful for risk stratification and developing more personalized treatment strategies. Deep muscle invasion is an important determinant of uterine cancer prognosis. A study by Dong et al. created a DL model to predict deep muscle invasion based on 4896 MR images from 72 EC patients and compared it to radiologist’s readings and achieved (90) an accuracy rate of 75%; nevertheless, the difference was not statistically significant. In a similar study, Chen et al. performed an analysis on 530 MR images and generated 84%, 66.7%, and 87.5% accuracy, sensitivity, and specificity, respectively (91). Lymph node metastasis (LNM) is one of the strong predictive factors for EC (88)]. Xu et al. developed a prediction model for LNM of normal size using MR images and CA125 values from 200 specimens of EC patients. The result showed approximately 85% accuracy (92). Recently, endometrial cytology has been reported as a viable diagnostic tool for detecting EC with good sensitivity and specificity (93, 94)]. A study was performed by Markis et al. in which they aimed to develop an automatic diagnostic system to analyze liquid endometrial cytology images of 416 patients using DL and found 90% accuracy for this model (95). Collectively, these studies suggest that ML has made remarkable progress in EC care.

### ML Model for Classifying Endometrial Lesions

Hysteroscopy for endometrial lesions is one of the gold standard procedures in an examination of the endometrium. Hysteroscopy is used to differentiate uterine body tumors, such as endometrial polyps and EC, which however depends on the hysteroscopic expertise. It has certain limitations such as it is dependent upon the comprehensive knowledge and understanding of the target pathology, lesion size, penetration depth of the lesion, skills, and expertise of the physician, availability of equipment, and assessment of patient comorbidities (65). ML-aided approaches to examine EC not only increase accuracy but also provide a minimally invasive and less expensive tool to correctly diagnose EC. A study conducted by Zhang et al. developed a CNN-based computer-aided diagnosis system using the VGGNet-16 model for diagnostic hysteroscopy image classification (96). Using 1,851 hysteroscopic images of uterine patients as input, Zhang et al. also investigated the VGGNet-16 CNN model efficiency for the classification of endometrial lesions. The result showed 80.8% overall accuracy suggesting that the CNN model could be used as a tool for EC diagnosis.

### ML Model for Classifying DNA Mismatch Repair–Deficient ECs

Approximately 3% of ECs are caused by germline mutations in the MMR genes such as MLH1/2, MSH6, and PMS2 and are termed MMR-deficient (MMR-D) tumors (97, 98). Recently, a study by Veeraraghvan et al. used contrast-enhanced CT to identify DNA MMR-D and/or tumor mutational burden-high (TMB-H) subtypes in ECs (99). This study built two ML models using generalized linear regression (GLMNet) and recursive feature elimination random forest (RF) classifiers to effectively differentiate between low copy number or high copy number MMR-D in ECs and also increasing rate of TMB-H in ECs. The authors analyzed data from a cohort of 422 patients and prefiltering was performed using GLMNet. Their findings indicated that radiomic models using ML algorithms can be utilized as a reproducible complementary or companion diagnostics for clinical trial enrollment and standard-of-care treatment.

## ML Algorithms in EC Prognosis

Lately, ML algorithms have been utilized in cancer care with an aim to better understand cancer prognostication. The ability of ML-based algorithms to detect, predict, and identify cancer using complex datasets indicates their importance. Over the past decade, several ML algorithms have been widely applied to EC prediction and prognostication. In a study, Praiss et al. (85) adapted an unsupervised ML algorithm named Ensemble Algorithm for Clustering Cancer Data (EACCD) and used it to classify patients based on TNM [tumor (T), nodes (N), metastases (M)] staging, grade, and age. EACCD is a combination of clustering method that derives dissimilarity among two combinations by continuously applying criteria-based clustering, followed by combining the learned dissimilarity estimate with a hierarchical clustering approach to find ultimate clusters of combinations. This innovative ML method improved the prognostic prediction for EC (85, 100). In another study, Chen and colleagues developed a tool ESTIMATE (Estimation of STromal and Immune cells in MAlignant Tumors) that uses gene expression data to predict tumor content and the degree of infiltrating stromal/immune cells from tumor tissues (101). ESTIMATE is a reliable algorithm that is widely accepted and has been used to determine the immune and stromal scores in various cancers such as breast cancer (102), glioblastoma (103), prostate cancer, colon cancer (104), and cutaneous melanoma (105). ESTIMATE total scores were found to be substantially closely associated with tumor purity in clinical tumor samples and tumor cell line samples, and they offered a simple and effective method for determining the number of tumor cells in a sample. ESTIMATE could further be improvised by the inclusion of endothelial cell signatures and tumor type–specific normal epithelial cells.

Most women with early stages of EC show a good prognosis. However, among them, 15% of patients with stage I and II EC develop recurrence (106). A study done by Akazawa et al. utilized EC patients and applied five ML algorithms: RF (107), logistic regression (LR) (108), decision tree (DT) (109), support vector machine (SVM) (110), and boosted tree (111), for the prediction of recurrence based on multiple clinical parameters such as age, body mass index, stage, histological type, grade, surgical content, and adjuvant chemotherapy. To verify the effectiveness of these classifications, accuracy and area under the curve (AUC) were analyzed. The maximum accuracy was reported by the SVM followed by LR and the least by boosted trees. The best AUC was observed in LR and the least in RF. Hence, they reported LR as the best predictive model for the study (108). They demonstrated the feasibility of AI prediction in patients with EC through the current investigation and concluded that, in the early stage of EC, the application of an ML algorithm made it possible to predict recurrence (111, 112). This finding can help to improve the efficiency and accuracy to predict recurrence and treatment response.

Lymph node involvement (LNI) is a significant prognostic indicator for several cancers including EC. However, at present, there is no validated method to predict LNI accurately in EC. Recently, a study by Günakan et al. investigated the use of the Naïve Bayes (NB) algorithm (113) for LNI prediction in EC patients. This study used various histopathological factors such as final histology, presence of lymphovascular space invasion (LVSI), grade, tumor diameter, depth of myometrial invasion, cervical glandular stromal invasion, tubal or ovarian involvement, and pelvic LNI. The study reported that the algorithm predicted the LNI using histopathological factors with high accuracy and concluded that ML could occupy a position in decision-making for managing EC. Subsequent studies using sentinel lymph nodes (SLNs), biochemical data, or imaging combined with ML algorithms might assist in EC management (114). Another study by Reijnen et al. aimed at developing and validating externally a preoperative Bayesian network (BN) to predict LNM and disease-specific survival (DSS) in EC patients (115). The study included 763 patients, who had been treated surgically for EC. Using score-based ML, an externally validated ENDORISK- BN (116) was developed for EC patients involving the various molecular, histological, and clinical biomarkers. Both outcomes showed high discriminative performance and good calibration. With a marginal rate of false negative 1.6%, ENDORISK was able to identify more than 55% of the patients at 5% risk for LNM. This approach guides both the patient and the clinician regarding personalized risk assessments evaluating the needs of patients and also the scope of the surgical solution. The work also demonstrated how, by adding easily available and multimodal biomarkers, BN may be utilized to personalize therapeutic decision-making in oncology (115, 117).

Endometrioid endometrial adenocarcinoma (EEA) is the most common type of EC among all types. A poor prognosis for disease dissemination has been associated with a high tumor grade, late surgical stage, and LVSI (118). All of these characteristics suggest that traditional clinical features are insufficient to accurately predict EEA prognosis. Therefore, developing a predictive prognostic model for EEA would be of great clinical value. A study by Yin et al. developed a prognostic model for EEA that combines gene expression and traditional features using RF. Three models were derived using (a) 11 genes alone, (b) stage and grade, and (c) both 11 genes and stage and grade. The study reported that the RF model derived from the “11 genes and grade” performed better than RF models derived from the 11 genes or grade alone, indicating that the RF model derived from the “genes and clinical features” had a stronger predictive ability for the prognosis of EEA. Thus, a combined RF model and clinical criteria may serve better for the stratification of patients in the clinic (119–122).

### ML Models for EC Screening, Risk Prediction, and Classification

Hart et al. used ML algorithms to categorize patients as low, medium, or high risk (123). They evaluated the model’s performance on the three-tier risk classification to that of physicians’ judgment and found encouraging results. These models offer a non-invasive and a cost-effective method of identifying high-risk subpopulations which might gain from early EC screening ahead of disease development. They discovered a unique and successful technique for premature cancer diagnosis and preventative measures for individual patients by performing a statistical biopsy of personal health data. Predicated on publicly available personal health data, seven alternative models, namely LR, neural network (NN), SVM, DT, RF, linear discriminant analysis, and NB (124) were developed to estimate the likelihood of an individual woman having EC in 5 years. The RF model outperformed the other six models regarding AUC with the NN coming in second. Both models were employed in dividing the population into three risk groups *viz.* low-, medium-, and high-risk groups. It does not aid in choosing the most effective preventative approaches (e.g., diet and exercise, progestin or antiestrogen therapy, and insulin-lowering therapy). Nonetheless, the ML approach holds great promise for assisting in the early detection of EC, as it produces high-accuracy predictions based primarily on personal health information before disease onset without the need for any invasive or expensive procedures such as endometrial biopsy or TVUS (123). Establishing personalized cancer preventive measures for each patient might benefit from a risk prediction model which classifies the population between low-, medium-, and high-risk categories (125). A model like this can assist doctors to identify high-risk groups for whom they can recommend measures to reduce the risk of EC, including dietary and activity modifications, progestin or antiestrogen medication, insulin-lowering therapy, and scheduled endometrial biopsies.

### Prediction of EC Risk

According to a current analysis, establishing personalized cancer preventive measures for each patient might benefit from a risk prediction model which classifies the population between low-, medium-, and high-risk categories (125). A model like this can assist doctors to identify high-risk groups for whom they can recommend measures to reduce the risk of EC, including dietary and activity modifications, progestin or antiestrogen medication, insulin-lowering therapy, and scheduled endometrial biopsies.

### EC Risk Factors: A Statistical Meta-Analysis and the Use of Artificial Neural Network to Develop a Risk Prediction Model

Using a statistical meta-analysis technique, Hutt et al. aimed at establishing the rank order of risk factors for EC and generating a collective risk and per centum risk for each component, followed by creating a NN computer model that could predict whether a patient’s overall cancer risk would increase or decrease. The objective was to determine whether a patient should be tested, to predict diagnosis, and to advise preventative actions to patients. To quantify relative risk, a meta-analysis of available data was performed, followed by the design and implementation of a risk prediction computer model based on a NN algorithm. Data for the meta-analysis of EC patients with the risk factors were taken from National Cancer Institute (NCI). Using a statistical meta-analysis technique, they were able to identify the rank order of risk variables for EC which was used to generate a pooled risk and risk percentage for each factor. Furthermore, using a computer NN model system, they developed a model that could predict an overall increase or decrease in cancer risk and cancer diagnosis for specific patients with 98.6% accuracy. The findings of the study effectively reduce the amount of unnecessary invasive testing of EC patients. This might be a valuable tool for physicians to utilize in concert with additional indicators to determine whether individuals require enhanced preventative measures before the potential development of EC (126).

## Limitations of ML Approaches in EC

The sensitivity and specificity of EC data are frequently necessary to train ML-based models. Data access should be carefully controlled to protect privacy without impeding innovation and technological development to enhance performance. Some of the major challenges associated with the implementation of ML in EC datasets are as follows:

- Retrospective versus prospective studies: Recruitment of the bulk of patients in the presented studies has been retrospective using the past labeled data for the training and testing of the used algorithms. Through prospective studies only, may one infer the true utility of the built systems when utilized in the real world. The introduction of wearable technology is facilitating massive prospective trials of historical standards; for instance, a study to diagnose atrial fibrillation in 419,093 consenting Apple watch holders is currently taking place (127).
- Peer-reviewed randomized controlled trials as the gold standard: To develop trust and acceptance of ML amongst the medical community, peer-reviewed evidence plays a pivotal role. In addition to this, there are very few randomized controlled trials published to date. ML experiments require high-quality reporting. The probability of bias and the possible utility of prediction models can only be accurately measured if all facets of a diagnostic or prognosis model are fully reported (128).
- Metrics often do not reflect clinical applicability: Amid the widespread usage in ML research, the AUC of a receiver operating characteristic is usually not the strongest measure for representing clinical validity and is difficult for many clinicians to comprehend. Clinicians ought to be trained to determine how the proposed algorithms can enhance patient treatment in a real-world setting, but most articles do not attempt to do so; other methods have been proposed, such as decision curve review that attempts at measuring the total advantage by using a formula to direct future behavior (129–131).
- Difficulty in comparing different algorithms: Since each test’s output is recorded using various tools and methods on various samples with distinct sample distributions as well as characteristics, quantitative comparison of algorithms through studies is difficult. Algorithms must be compared on a similar self-dependent test set, which is depictive of the target population utilizing the comparable effectivity measures to produce fair comparisons. Lacking this, physicians would struggle to determine which algorithm is most likely beneficial for the patients’ (127).
- Dataset shift: The clinical and operational practices evolve, and the data are generated in a non-stationary environment amidst the shifting population of patients. When a novel predictive algorithm is introduced, it may induce shifts in operation, leading to a different distribution from the one used to generate the results. As a result, detection systems drift and update models about the quality of the results (132).
- Algorithmic bias: Clinical assessment should be performed on a representative sample of the planned implementation population, and algorithms should be constructed keeping the global community in mind. Factors such as age, race, sex, sociodemographic stratum, and position should be considered when analyzing output by population subgroups. Researchers should be guided to make sure that the proper measures are taken to assess bias while adopting models as there is a better understanding of these problems, and clinicians are empowered to engage objectively in system design and growth. Algorithm bias may be divided into three inputs: (1) bias of the model (models chosen to best reflect the major, not exclusively underrepresented groups), (2) variance of the model and model ambiguity (owing to a lack of data from minorities), and (3) noise in the results (the impact of a collection of unknown parameters on model predictions, which can be avoided by selecting subpopulations in which to test supplementary variables) (127).
- Challenges in generalization to new populations and settings: External validation, which involves evaluating an AI system with accurately scaled datasets obtained from organizations except for those that supplied the statistical model training, is required for the accurate evaluation of real-world clinical output and generalizability. This will guarantee that any important changes in target patient demographics and conditions of illness in real-world clinical settings are appropriately reflected in the system where it will be used. This technique is currently uncommon in the literature and is a major source of concern. A current systematic assessment of research that assessed AI algorithms for medical imaging diagnostic analysis discovered that only 6% of 516 relevant scientific publications completed external validation (133, 134).
- Logistical issues in implementing AI systems: The reality that almost all medical data are not widely accessible for ML is causing several of the existing difficulties in applying AI to clinical research. Data are often segmented in a variety of medical imaging archival programs, diagnostic systems, EHRs, automated monitoring software, and insurance databases, making it impossible to integrate (135, 136).
- Human obstacles to AI adoption in healthcare: A good understanding of human and computer interactions should be a focus as there are significant human obstacles to adoption. It will be critical to retain an emphasis on clinical applicability and medical outcomes to make sure that this technology reaches and benefits the individual (127).

Also, other key points to be addressed are that the input data should be of good quality, with few artifacts or noise levels. While ML models might handle noisy input to a certain measure, incorrect labels may significantly affect an ML model’s performance. Similar to different statistical approaches, many ML models require a training set with no missing characteristics, so the training sets must be as thorough as feasible. While data augmentation approaches, ranging from random imputations to ever further complex ML-based algorithms, could be implemented to substitute the missing data, they typically do not produce the identical results as utilizing a complete dataset for training. Furthermore, bigger datasets are typically desired since they allow the ML model to understand the real variance in the data with a lower chance of small outliers negatively affecting the model.

## Future Perspective

**Figure 3:**
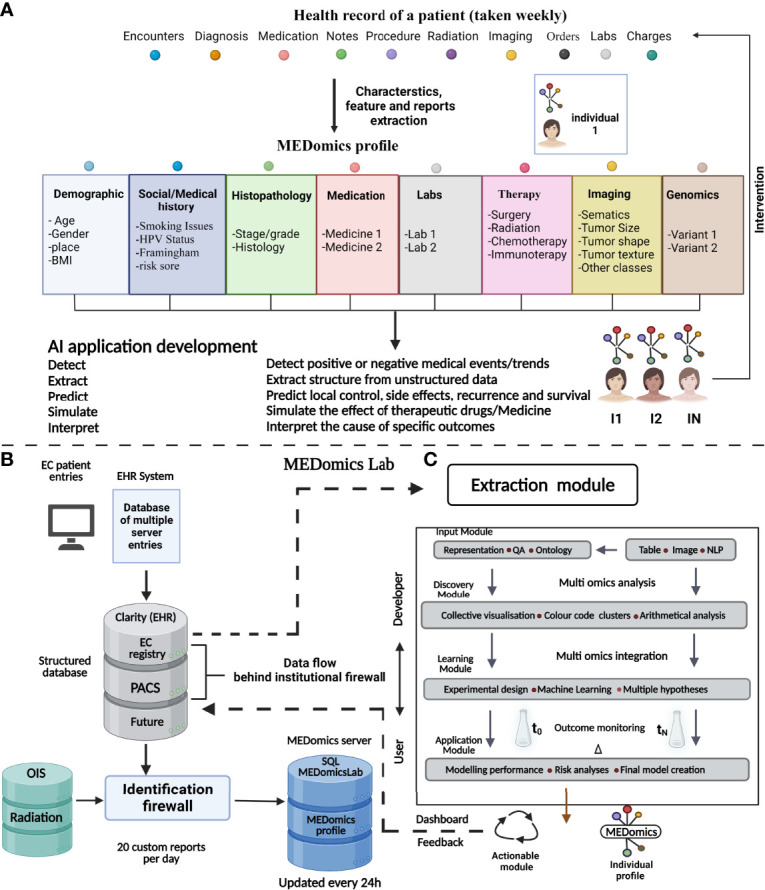
The MEDomics approach for EC prediction and prognostication. An AI framework integrating EHRs with continuous learning infrastructure called “MEDomics” could use multimodal clinical data comprising thousands of cancer patients and millions of data points. The data are automatically extracted and integrated with analytical workflows. In addition, integration of natural language processing models is utilized for extraction of medical notes and classifying patients into different risk groups. The system has the potential to develop hypotheses based on the patients’ data rather than laboratory data alone and can help to choose disease therapy and monitor disease prognosis. **(A)** Illustration of a patient’s electronic health data records over a normal cancer care timeline. The MEDomics patterns are then used to construct AI and medical informatics software utilizing an “*in silico* randomized clinical trial” technique to discover, retrieve, anticipate, model, and evaluate important clinical endpoints. Management dashboards and other types of communication between health practitioners and data scientists are eventually used to transmit actionable solutions back into the health system. **(B)** Flow of medical data for the construction of AI and the building of MEDomics profiles. The EHRs and the Clarity relational database keep track of medical actions in real time. Clarity generates custom reports, which are then transmitted to the MEDomics server for deidentification, data structuring, and extraction/calculation of features. Data are eventually collected and refreshed every day on the MEDomics database installed behind the institutional firewall which is protected for access *via* a double identification. **(C)** MEDomics Lab as a system for medical research. Health records from institutional systems may be fed into a structured dataset, which is then fed into the MEDomics Lab system. The MEDomics Lab engine combines and processes these data, which is then employed in five separate computing modules: input, extraction, discovery, learning, and application. As a result, statistical models for precision oncology are developed, which may then be returned to hospital databases to aid clinical decision-making. QA, quality assurance; BMI, body mass index; HPV, human papillomavirus; PACS, picture archiving and communication system; OIS, oncology infrastructure system; *SQL*, Structured Query Language.

An electronic health record (EHR) includes data comprising the physician’s notes and other information documenting a patient’s clinical history (137). The use of clinical data in research is challenging due to several reasons: in that, raw clinical data are often variable and are not well annotated, the clinical data are accumulated in an unlinked manner making it complex for research application, and the multimodal (i.e., radiology images, physician notes, pathology images, and laboratory results) nature of the data makes it hard to be automatically analyzed by ML algorithms without prior data curation by human intervention (138). Therefore, introducing a novel data integration and decision support system intended to harness the potential of EHRs for EC management might be an attractive addition to the computer-guided EC research (139) (**Figure 3**). An information technology infrastructure should be established to ease the creation and testing of statistical learning models for EC. This development linked multiple organizational EHR database systems and continually gathers data in a safe, comprehensive, and extendable manner. MEDomics profiles of EC patients, which are at the heart of the conceptual methodology, are synthesized data structures that encompass a specific EC patient’s full clinical service chronology. The longitudinal EC patient’s profiles are then utilized for a variety of AI application designs, to communicate meaningful interventions back into the health system. EC patient’s cohorts were identified and were used to examine institution-wide mortality outcomes among EC patients along with examining the efficiency of targeted therapy and overall survival.

**Figure 4:**
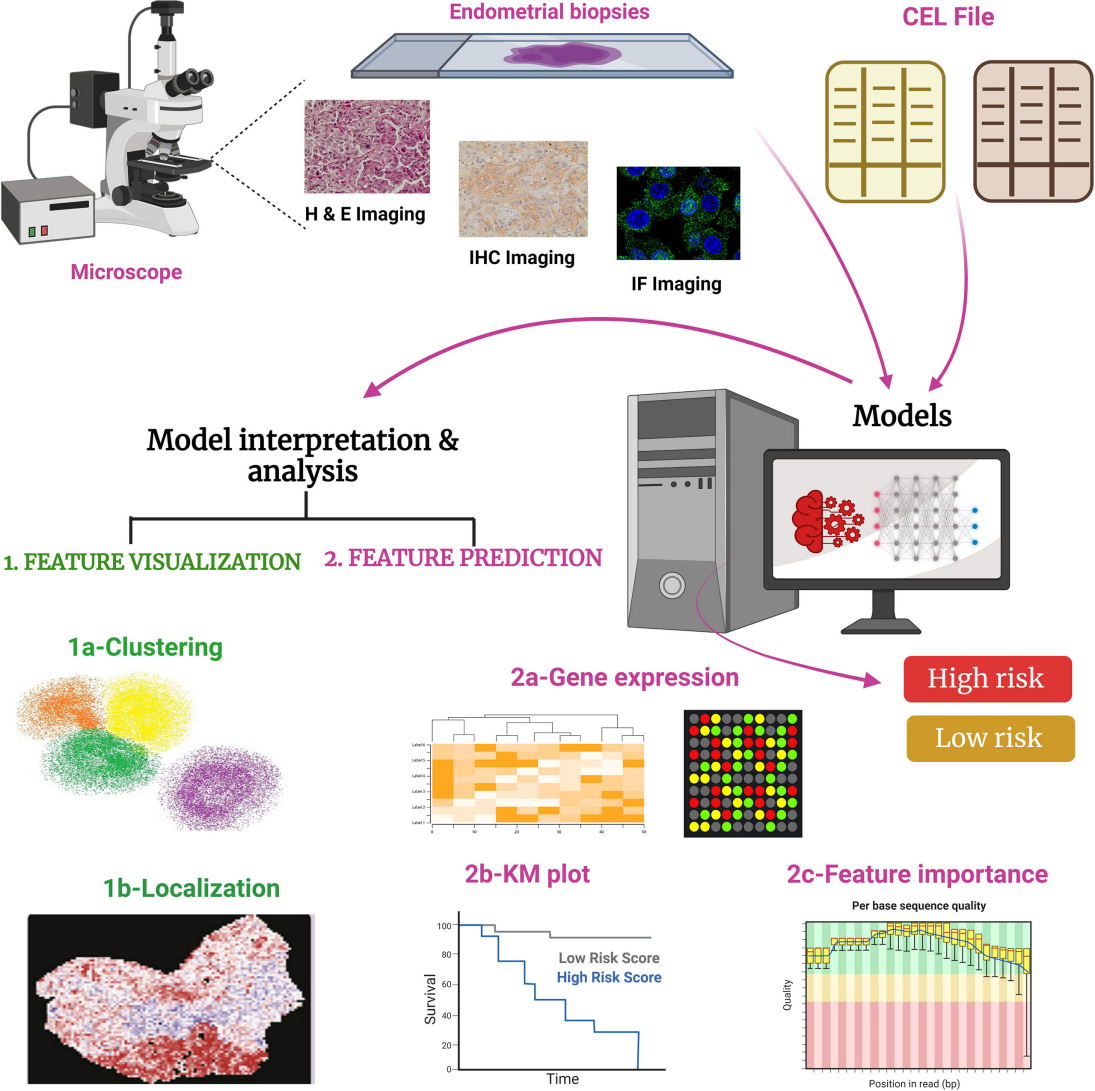
Working of PANOPTES, a multiresolution deep convolutional neural network (CNN). In this trained model generated through the CNN algorithm, pathological images were used to predict the gene mutations and histological and molecular subtypes of EC. PANOPTES ML models using multiresolution architecture can classify histological subtypes of EC, molecular subtypes, and critical mutations (loss or gain of functions) with decent performance based on H&E (hematoxylin and eosin), IHC (immunohistochemistry), and IF (immunofluorescence) images. Also, some data can be accessed from the input CEL files. Predicated on the input, CNN models identify subtypes and mutations in EC. Multiresolution CNN models outperform single-resolution CNN models on the visual patterns. CNN models would incorporate human interpretable tumor characteristics according to feature extraction. Tumor grade identifies the molecular subtype and classifies into high-risk or low-risk cohorts from endometrioid histology samples to capture characteristics of varied sizes on the H&E, IHC, and IF slides, which is similar to a human operator pathological evaluation. Unlike traditional CNN architectures, Panoptes’ input is a group of three tiles from the same region on the image slide rather than one tile. CEL file: differential expression profile generated by Affymetrix DNA microarray-based software analysis.

Cancer genotype determination has garnered increased attention to take advantage of biomarker-based targeted therapies (140). Molecular diagnostics have shown promising results in determining genetic biomarkers that are determined by molecular assays but molecular assays are time-consuming and are not available to all patients (140). Developing a link between genotype and phenotype seems a promising approach. Recent studies have shown that AI-based histologic diagnosis links histology, molecular biomarkers, and prognosis in cancer (141, 142). In a recent development in EC diagnosis, a multiresolution deep CNN named “Panoptes” was utilized where pathological images were used to predict the gene mutations and histological and molecular subtypes in EC (**Figure 4**) (143). The model was able to read one slide in 4 min and could predict 18 common gene mutations without sequencing analysis, providing a cost-effective cancer detection method. It is anticipated that the multiplex diagnosis and prognosis of different cancer types could be developed where a model trained for one cancer type might apply to other relevant cancers.

Minimal invasive sampling procedures aid molecular studies to help in detection and prevention of EC. More frequent tests or more different tests would almost likely assist early detection approaches (among the high risk or symptomatic); however, the potential value of screening among the asymptomatic must also be evaluated. Molecular testing may aid in refining present diagnostic algorithms among symptomatic women by reducing the effectiveness and rejection frequency of histological diagnosis that now restricts the effectiveness of endometrial aspirate–based diagnosis (144). Various screening techniques are being developed to aid the early detection of EC; one such developed test is the PapSEEK test which recognized the majority of women having EC and one-third of women having ovarian cancer in the NCI-funded study that employed the Pap test (standard screening test) among women who had previously been confirmed with cancer (145). SLN operations are becoming increasingly common in the management of EC, and the performance of SLN biopsy and the positive effect of early-stage EC were evaluated in a clinical trial showing that the procedure was feasible and safe. SLN mapping is built upon the idea of LNM as a consequence of a systematic process. The lymph empties in a precise manner away from the tumor; hence, when the SLN, or initial node, is negative for metastasis, the nodes following the SLN would likewise be negative. While the disease needs to be properly staged to ensure an accurate prognosis and a selection of suitable treatment strategies, such techniques would assist patients to escape the adverse consequences involved in a total lymphadenectomy. Surgical expertise, commitment to an SLN methodology, and the utilization of pathological “ultra-staging” are all important variables in SLN mapping effectiveness (146, 147)

Although ML techniques can learn from both small and large datasets, the size and homogeneity of the datasets used for ML or DL models are equally important for the accuracy of the models. Most of the AI models are trained on small training datasets resulting in a compromised accuracy of the model. Generating larger datasets from real-world samples is a daunting task. Although challenging, the lab-on-a-chip and organ-on-a-chip technologies can be explored to simulate the tumor microenvironment and generate clinically relevant larger datasets required to develop powerful algorithms and models. Since clinical data (the major source of cancer research) sharing is a pressing challenge due to ethical and legal issues, efforts can be made to develop a secured data sharing system. For instance, a learning system can be developed where the raw data remains with the source organization or institution, and a model is shared with the clinic to preprocess the raw data, and then the processed/curated data are to be shared with the research center (transfer learning making a web of information sharing in a protected way). In this regard, robust AI systems with smart strategies are needed. It is envisaged that, in near future, AI-based continuous learning could develop a smart decision-making system where both the physician and patient could be benefited.

## Author Contributions

VB, AS, and SP prepared the first draft of the manuscript and editing. IG and XZ, editing. PEL, PQ, and VP, conceptualization, writing, reviewing, and editing. All authors contributed to the article and approved the submitted version.

## Funding

This work was also supported by the Shenzhen Key Laboratory of Innovative Oncotherapeutics (ZDSYS20200820165400003) (Shenzhen Science and Technology Innovation Commission), China, Shenzhen Development and Reform Commission Subject Construction Project ([2017]1434), China, Guangdong Basic and Applied Basic Research Foundation (2019A1515110970), China, Overseas Research Cooperation Project (HW2020008) (Tsinghua Shenzhen International Graduate School), China, Universities Stable Funding Key Projects (WDZC20200821150704001), TBSI Faculty Start-up Funds, China and The Shenzhen Bay Laboratory, China.

## Conflict of Interest

The authors declare that the research was conducted in the absence of any commercial or financial relationships that could be construed as a potential conflict of interest.

## Publisher’s Note

All claims expressed in this article are solely those of the authors and do not necessarily represent those of their affiliated organizations, or those of the publisher, the editors and the reviewers. Any product that may be evaluated in this article, or claim that may be made by its manufacturer, is not guaranteed or endorsed by the publisher.
